# Suboptimal Herd Performance Amplifies the Spread of Infectious Disease in the Cattle Industry

**DOI:** 10.1371/journal.pone.0093410

**Published:** 2014-03-26

**Authors:** M. Carolyn Gates, Mark E. J. Woolhouse

**Affiliations:** Epidemiology Group, Centre for Immunity, Infection and Evolution, School of Biological Sciences, University of Edinburgh, Ashworth Laboratories, Edinburgh, Scotland, United Kingdom; The University of Tokyo, Japan

## Abstract

Farms that purchase replacement breeding cattle are at increased risk of introducing many economically important diseases. The objectives of this analysis were to determine whether the total number of replacement breeding cattle purchased by individual farms could be reduced by improving herd performance and to quantify the effects of such reductions on the industry-level transmission dynamics of infectious cattle diseases. Detailed information on the performance and contact patterns of British cattle herds was extracted from the national cattle movement database as a case example. Approximately 69% of beef herds and 59% of dairy herds with an average of at least 20 recorded calvings per year purchased at least one replacement breeding animal. Results from zero-inflated negative binomial regression models revealed that herds with high average ages at first calving, prolonged calving intervals, abnormally high or low culling rates, and high calf mortality rates were generally more likely to be open herds and to purchase greater numbers of replacement breeding cattle. If all herds achieved the same level of performance as the top 20% of herds, the total number of replacement beef and dairy cattle purchased could be reduced by an estimated 34% and 51%, respectively. Although these purchases accounted for only 13% of between-herd contacts in the industry trade network, they were found to have a disproportionately strong influence on disease transmission dynamics. These findings suggest that targeting extension services at herds with suboptimal performance may be an effective strategy for controlling endemic cattle diseases while simultaneously improving industry productivity.

## Introduction

Beef and dairy herds require a constant supply of replacement breeding cattle to maintain or increase herd size. A key decision facing producers is whether to raise heifers internally for replacement or to purchase replacement breeding cattle directly from outside sources at the risk of introducing many economically important diseases [1]–[5]. The optimal strategy for any given herd depends on a number of complex factors including land and labour availability, cash flow needs, market prices, and future business goals [6]–[10]. Heifers require intensive management and nutritional support to reach an appropriate physical maturity by the target age at first breeding [11] and for farms that cannot provide this cost-effectively, there can be significant financial advantages to breeding calves with desirable growth and carcass characteristics for fattening instead [12], [13]. Due to the long production cycle of cattle, farms that are undergoing rapid expansion to capture favourable market prices may also choose to purchase replacement cattle rather than rely on internal growth [14].

In some cases, however, the decision to purchase replacement cattle is directly influenced by herd reproductive performance. Farms that cull excessive numbers of animals for infertility, poor production, and other health related issues have an increased demand for replacement breeding cattle [15], while farms with high calf mortality rates, delayed ages at first calving, and prolonged calving intervals may not have an adequate supply of heifers to meet replacement needs [16], [17]. As well as losing significant profit through reduced productivity [17], [18], these farms are potentially increasing their risk of disease introductions by purchasing greater numbers of replacement breeding cattle than would be needed if they were achieving industry standards for performance. Since the movements of replacement breeding cattle form part of a larger contact network, herds that purchase large numbers of animals to compensate for poor performance may also be contributing to the industry-level transmission dynamics of many infectious cattle diseases.

Although there have been many recent studies characterizing the frequency of between-herd cattle movements and the basic structure of cattle movement networks in countries with electronic movement recording systems [19]–[26], little is currently known about the underlying causes or epidemiological consequences of trade in replacement breeding cattle. In this analysis, data from the national cattle movement database in Great Britain was used as a case example to determine the relationship between key herd performance indicators (average age at first calving, interval between successive calvings, culling rates, and calf mortality rates) and the number of replacement breeding cattle purchased by beef and dairy herds. Simple disease simulation models were then used to study the effects of removing replacement breeding cattle movements from the contact network on the transmission dynamics of different endemic pathogens. Findings from both analyses were used to emphasize that the management decisions of individual herds can have a substantial impact on the epidemiology of infectious disease at the industry level.

## Materials and Methods

### Cattle movement data

Farmers across the European Union have been required to report the births, deaths, and movements of individual cattle to the government under Council Regulation (EC) No 820/97 as part of efforts to restore consumer confidence in the safety of livestock following the bovine spongiform encephalopathy (BSE) crisis in 1996. In Great Britain, these records have been stored electronically in the Cattle Tracing System (CTS) database operated by the British Cattle Movement Service (BCMS) since January 2001 [27], [28]. Demographic information on the sex, breed classification (beef, dairy, or dual purpose breed), date of birth, birth location, date of death, death location, and identity of calves that survived parturition is also available for each animal and may be used to generate key performance indicators for cattle breeding herds [29]. Movements on or off livestock locations are recorded with information on the departure location, destination location, movement date, and movement type (birth, death, or movement). By linking the demographic information with the movement records, it is possible to infer the animal's production purpose at the time of movement.

The subsequent analyses used data from 01 January 2004 through 31 December 2006 to characterize the performance of British cattle farms and to reconstruct the network of cattle movements between them. Data were extracted from the CTS database using the Python programming language. For the purpose of this analysis, a farm was defined as any location with a unique county-parish-holding (CPH) number that was classified as an agricultural holding or landless keeper (farmer raising cattle on rented land). The primary reason for selecting this time period was to ensure that sufficient pre- and post-movement data was available to classify animals into production groups. It was assumed that animals intended for human consumption would be slaughtered by 30 months of age to comply with bovine spongiform encephalopathy (BSE) regulations [30] and that animals intended for breeding would deliver their first calf by 48 months of age. At the time of this analysis, CTS data was only available through April 2010.

### Herd performance indicators

There were a total of 8,415,283 recorded calvings on 67,868 farm locations in Great Britain from January 2004 through December 2006. This analysis focused on the subset of 34,289 farms with an average of at least 20 beef and/or 20 dairy cattle births per year. This included 18,951 exclusively beef farms, 14,737 exclusively dairy farms, and 601 mixed production farms. Altogether these herds accounted for 89.6% of the total number of calvings in Great Britain. The main reasons for restricting the sample were to eliminate small scale operations where cattle breeding was unlikely to be the primary source of farm income [31] and to eliminate farms that may have been in the process of entering or exiting the cattle industry. Beef herds and dairy herds managed on mixed production farms were treated as separate units in the remaining analyses.

For each calving event, the following information was recorded: calving farm, calving date, dam date of birth, dam breed classification, date and location of any previous or subsequent calvings, date of the next recorded movement off the calving farm, calf breed classification, calf sex, and calf date and location of death. The average number of calvings per year was used as an estimate of breeding herd size. The basic calving event records were aggregated by farm to generate the following performance indicators: average age at first calving, calving interval, culling rate, and calf mortality rate. The methodology used to calculate these indicators has been published in other studies [29], [32].

The average age at first calving was calculated as the difference between the age at calving and date of birth in months for all heifers that calved on the farm during the specified time period. A heifer was defined as an animal between 19 and 48 months of age with no previously recorded calving dates in the CTS database. The purpose for placing restrictions on age was to eliminate potential outliers that may have been caused by data entry errors or animals that may have delivered an unrecorded stillborn calf at an appropriate age. The calving interval was calculated as the number of months between successive calving dates for the subset of dams that delivered another calf within 730 days. It was assumed that in most production herds, any animals that failed to deliver a calf within 24 months would be culled from the herd and outlying values were most likely attributable to data entry errors or unrecorded births. The culling rate was calculated as the percentage of calvings where the dam was subsequently slaughtered or sold within 500 days of calving. The calf mortality rate was calculated as the percentage of all calves born during the specified time period that died on an agricultural holding within 365 days of birth. It was assumed that calves slaughtered at an abattoir were intended for the veal production market and therefore excluded from the mortality calculations. The performance indicators were averaged over the 3 year study time period.

### Network reconstruction

There were a total of 7,917,890 individual movements between cattle farms in the period from 01 January 2004 through 31 December 2006. The cattle movement network was reconstructed by aggregating the individual movement records into batch movement records such that all cattle moving from farm A to farm B on the same date were considered a single batch movement. This resulted in a network with 2,695,402 batch movements between 90,478 unique farm locations (including breeding herds, fattening herds, and hobby farms). Similar to previous studies, movements that occurred through a livestock market were treated as a single direct movement from the original departure herd to the final destination herd after sale [20], [33]. Approximately 1% of individual movement records were discarded due to missing or inaccurate information.

For the purpose of this analysis, a replacement breeding heifer was defined as an animal that was born on a different location than the destination farm and subsequently calved on the destination farm, while a replacement breeding cow was defined as an animal that previously calved on a different location than the destination farm and subsequently calved on the destination farm. These definitions were used to distinguish true cattle sales from temporary movements between seasonal grazing pastures, movements between locations operated by the same cattle business, and movements through farms acting as livestock dealers. All batch movements that contained at least one replacement breeding female were subsequently classified as replacement breeding cattle movements. The remaining movements included store calves, fattening cattle, breeding bulls, and replacement heifers that were culled before breeding. The average number of replacement breeding cattle purchased by the study farms each year was also recorded.

### Descriptive statistics

Basic descriptive statistics on the performance of beef and dairy herds were provided as frequency distributions. For illustrative purposes, the industry standards for performance were also indicated on the plots. In general, it is held that the average age at first calving should be less than 24 months, the average calving interval less than 365 days, the average culling rate for beef herds between 15 and 20%, the average culling rate for dairy herds between 25 and 35%, and the average calf mortality rate less than 5% [7], [34]–[36].

### Impact of herd performance on replacement breeding cattle trade

Zero-inflated negative binomial (ZINB) regression models were used to explore the relationship between herd performance and the purchase of replacement breeding cattle. Data for beef herds and dairy herds were analysed separately due to inherent difference in management practices. The logistic component of the ZINB model provided insight on factors influencing the odds of herds remaining closed over the three year study period, while the negative binomial component provided insight on factors influencing the expected count of replacement cattle purchased over the three year study period. Prior to analysis, a logarithmic transformation (base 10) was applied to herd size, and the performance variables (age at first calving, calving, interval, culling rate, and calf mortality rate) were divided into categories by quintile. For culling rate, the reference category was set as the middle quintile and for the remaining variables, the reference category was set as the top quintile.

As the purpose of the analysis was to explore the relationship between performance indicators and replacement breeding cattle trade rather than to generate the most parsimonious model, all variables were retained in both the logistic and negative binomial components of the final multivariate models. The Vuong test statistic was used to confirm the choice of a zero-inflated model over standard negative binomial regression. The odds ratios (ORs) and 95% confidence intervals were reported for the logistic components of the models, while the coefficients and standard errors (SEs) were reported for the negative binomial components of the models. All statistical analyses were performed in R [37].

The equations from the final ZINB regression models were then used to predict the effects of improving herd performance on the total number of replacement breeding cattle purchased by beef and dairy herds. As a baseline for comparison, we first used the empirically observed values for the performance indicators in the model equations to estimate the total number of replacement breeding cattle purchased. Then, each of independent variables (with the exception of herd size) was set to a target value and the new predicted values for the total number of replacement breeding cattle purchased were calculated. For age at first calving, calving interval, and calf mortality variables, the target values were set as the top quintile for performance. For culling rate, the target values were set as the middle quintile for performance. These quintiles were used as the target performance levels based on observations that the majority of British beef and dairy were failing to achieve industry standards for performance in practice. The objective was to provide a more realistic estimate for how much performance could be improved. Each variable was tested alone and in combination. The results were expressed as the percentage reduction in the total number of purchased replacement breeding cattle compared to the baseline value.

### Impact of replacement breeding cattle trade on disease transmission dynamics

The effect of removing replacement breeding cattle movements from the contact network on disease transmission dynamics was evaluated with a simple Susceptible-Infectious-Susceptible (SIS) simulation model. At the beginning of each simulation, disease was seeded on 10,000 farms at random on 01 January 2004. Each affected farm was assigned an infectious period drawn at random from an exponential distribution with a half-life, *h* [2]. The model was then updated in time steps of one day. If an infected farm moved a batch of cattle to a susceptible farm, there was a fixed probability, *p*, that the destination farm would also become infected. The probability was not weighted according to the number of cattle moved. Farms that reached the end of their infectious period reverted back to a susceptible state. To ensure adequate time for the system to reach steady state equilibrium, the simulation was allowed to run for a total of 50 years by recycling the 3 year movement data set. Endemic prevalence was measured as the average number of farms infected on any given day over the last 3 years of the simulation. The simulation code was implemented in the C programming language.

In the first set of simulation scenarios, *h* was set at 1,095 days and *p* was set at 0.05 to approximate the transmission dynamics of a pathogen similar to bovine viral diarrhoea virus [2]. A targeted removal approach was used to assess the relative importance of replacement breeding cattle movements to network transmission dynamics [38]. At the beginning of each simulation, a proportion of replacement breeding cattle movements were removed from the network data set at random. The simulation was then run on the reduced movement network to monitor changes in the predicted endemic prevalence. A total of 10,000 simulations were performed with the proportion to be removed drawn at random from a uniform distribution bounded at 0 and 1 representing no removal and complete removal, respectively. Based on performance curves, this number of simulations was adequate to capture the variation in model outcomes. As a benchmark for comparison, another 10,000 simulations were performed where equivalent numbers of movements (including replacement breeding cattle movements and all other types of movements) were removed from the network at random. The results from both simulation sets were plotted as the percent of total network movements removed against the percent change in endemic prevalence using the maximum recorded value for endemic prevalence amongst the simulations as the baseline value.

In the second set of simulation scenarios, the proportion of replacement breeding cattle movements removed from the network was fixed at 1, but the values for *h* and *p* were varied in each replicate to determine whether the observed effects were consistent across for broader range of endemic pathogens. At the beginning of each simulation, the value for *h* was drawn from a uniform distribution ranging from 90 days to 1,825 days and the value for *p* was drawn from a uniform distribution ranging from 0.01 to 0.25. These parameter ranges were chosen based on how the simulated diseases behaved on the networks. Pathogens with farm infectious periods below 90 days were generally unable to persist. When the farm infectious period was increased above 1,825 days or the transmission probability was increased above 0.25, the network saturated and there was very little change in the endemic prevalence. A total of 100,000 simulations were performed. Similar to the first scenario, another 100,000 simulations were performed removing the equivalent number of movements (including replacement breeding cattle movements and all other types of movements) at random for comparison. The results were again expressed as the additional percentage change in endemic prevalence relative to the baseline simulations with random elimination of cattle movements.

## Results

### Descriptive statistics

**Figure 1:**
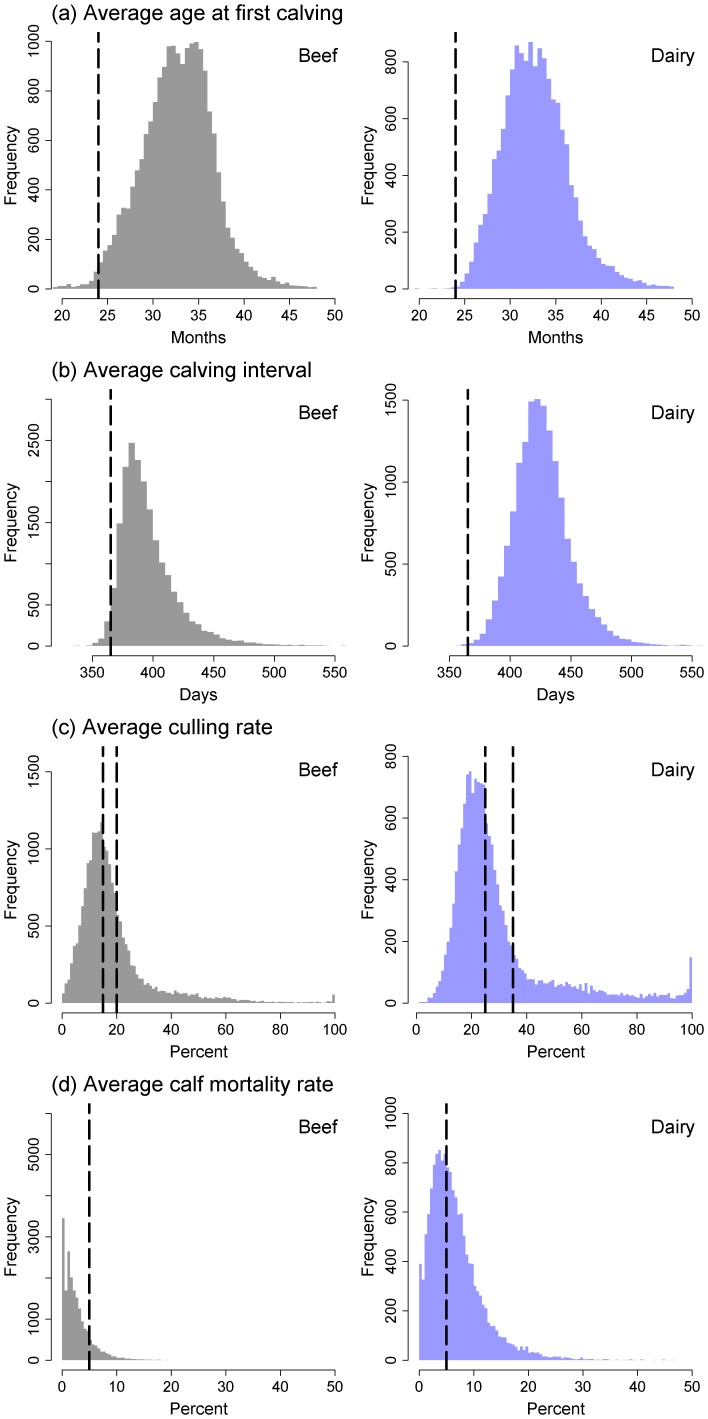
Descriptive statistics on the performance of beef and dairy herds in Great Britain. Frequency distributions of the (a) average age at first calving, (b) average calving interval, (c) average culling rate, and (d) average calf mortality rate amongst 24,093 beef herds and 14,754 dairy herds in Great Britain with at least 20 breeding dams per year between January 2004 and December 2006. The vertical dashed lines indicate the industry target values for performance.

Data on the performance of 19,552 beef herds and 15,338 dairy herds in Great Britain with an average of at least 20 calvings per year were derived from records stored in the national Cattle Tracing System (CTS) database between January 2004 and December 2006. The average size of beef herds in the sample was 56 breeding cattle (median: 41, range: 20 to 1,520) and the average size of dairy herds was 91 breeding cattle (median: 76, range: 20 to 1,241). As highlighted in Figure 1, there were a substantial number of herds performing below industry targets for average age at first calving, calving interval, culling rates, and calf mortality rates. An estimated 69% of beef herds and 59% of dairy herds purchased at least one replacement breeding animal over the three year period. The average number of replacement breeding cattle purchased by open beef herds in a given year was 6 (median: 2, range: 1 to 422), while the average number of replacement breeding cattle purchased by open dairy herds in a given year was 9 (median: 2, range: 1 to 847).

### Impact of herd performance on replacement breeding cattle trade

Zero-inflated negative binomial (ZINB) regression models were constructed to explore the relationship between herd performance and the total number of replacement breeding cattle purchased by beef and dairy herds (Tables 1 and 2, respectively). The Vuong tests for beef (V = 11.36, p<0.001) and dairy (V = 11.43, p<0.001) herds had high positive values indicating that the zero-inflated models fit the data better than standard negative binomial regression.

**Table 1:** Zero-inflated negative binomial regression model for beef herds.

		(a) logistic			(b) negative binomial		
Predictor	Levels	OR	95% CI	p-value	Coef	SE	p-value
log_10_(herd size)	–	0.85	0.67–1.09	0.201	2.12	0.037	<0.001
Average age at first calving (months)	<29.5	Ref	-	-	Ref	-	-
	29.6 to 31.8	0.47	0.37–0.59	<0.001	0.063	0.030	0.038
	31.9 to 33.8	0.45	0.36–0.56	<0.001	0.082	0.030	0.006
	33.9 to 35.8	0.89	0.74–1.06	0.194	−0.012	0.031	0.695
	>35.9	1.33	1.13–1.58	0.001	−0.126	0.033	<0.001
Calving interval (days)	<378	Ref	-	-	Ref	-	-
	379 to 386	0.78	0.64–0.95	0.012	−0.005	0.031	0.881
	387 to 396	0.76	0.63–0.93	0.007	0.001	0.031	0.992
	397 to 411	0.73	0.59–0.89	0.002	−0.009	0.031	0.776
	>412	1.16	0.97–1.40	0.102	0.091	0.032	0.005
Culling rate (%)^a^	<9.8	2.10	1.67–2.63	<0.001	−0.059	0.030	0.066
	9.9 to 13.5	1.55	1.23–1.96	<0.001	−0.043	0.030	0.153
	13.6 to 17.2	Ref	-	-	Ref	-	-
	17.3 to 23.4	1.24	0.98–1.57	0.071	0.164	0.030	<0.001
	>23.5	1.95	1.57–2.42	<0.001	0.667	0.032	<0.001
Calf mortality rate (%)^b^	<0.68	Ref	-	-	Ref	-	-
	0.69 to 1.52	0.88	0.72–1.07	0.199	0.067	0.032	0.038
	1.53 to 2.59	0.90	0.74–1.09	0.265	0.105	0.032	0.001
	2.60 to 4.29	0.80	0.66–0.98	0.033	0.121	0.032	<0.001
	>4.30	0.78	0.64–0.95	0.014	0.345	0.031	<0.001

The (a) logistic and (b) negative binomial components of the zero-inflated negative binomial regression model predicting the likelihood of being a closed herd and the number of replacement breeding cattle purchased by beef herds, respectively. (OR  =  odds ratio, CI  =  confidence interval, Coef  =  coefficient, SE  =  standard error)

Voung test V = 11.36, p<0.001

a The culling rate was calculated as the percentage of calvings where the dam was subsequently slaughtered or sold within 500 days of calving.

b The calf mortality rate was calculated as the percentage of all calves born during the specified time period that died on an agricultural holding within 365 days of birth.

**Table 2:** Zero-inflated negative binomial regression model for dairyherds.

		(a) logistic			(b) negative binomial		
Predictor	Levels	OR	95% CI	p-value	Coef	SE	p-value
log_10_(herd size)	–	0.90	0.73–1.11	0.333	1.707	0.051	<0.001
Average age at first calving (months)	<29.8	Ref	-	-	Ref	-	-
	29.9 to 31.7	0.62	0.52–0.74	<0.001	0.043	0.043	0.317
	31.8 to 33.5	0.81	0.69–0.95	0.012	0.172	0.044	<0.001
	33.6 to 35.6	0.93	0.80–1.09	0.398	0.167	0.045	<0.001
	>35.7	1.03	0.88–1.21	0.674	0.341	0.045	<0.001
Calving interval (days)	<408	Ref	-	-	Ref	-	-
	409 to 420	0.89	0.76–1.04	0.143	0.043	0.045	0.330
	421 to 429	0.89	0.75–1.04	0.138	0.005	0.045	0.907
	430 to 443	0.90	0.77–1.06	0.197	0.040	0.045	0.374
	>444	0.92	0.78–1.08	0.301	0.150	0.045	0.001
Culling rate (%)^a^	17.5	1.74	1.48–2.05	<0.001	−0.273	0.046	<0.001
	17.6 to 21.8	1.19	1.01–1.40	0.043	−0.199	0.043	<0.001
	21.9 to 26.4	Ref	-	-	Ref	-	-
	26.5 to 35.9	0.78	0.66–0.94	0.007	0.273	0.041	<0.001
	>36.0	1.41	1.20–1.66	<0.001	0.462	0.044	<0.001
Calf mortality rate (%)^b^	<2.86	Ref	-	-	Ref	-	-
	2.87 to 4.69	0.87	0.75–1.02	0.078	−0.029	0.047	0.538
	4.70 to 6.74	0.78	0.67–0.91	0.001	0.104	0.047	0.025
	6.75 to 9.80	0.59	0.50–0.69	<0.001	0.244	0.046	<0.001
	> 9.81	0.46	0.54–0.54	<0.001	0.376	0.046	<0.001

The (a) logistic and (b) negative binomial components of the zero-inflated negative binomial regression model predicting the likelihood of being a closed herd and the number of replacement breeding cattle purchased by dairy herds, respectively. (OR  =  odds ratio, CI  =  confidence interval, Coef  =  coefficient, SE  =  standard error)

Voung test V = 11.43, p<0.001

a The culling rate was calculated as the percentage of calvings where the dam was subsequently slaughtered or sold within 500 days of calving.

b The calf mortality rate was calculated as the percentage of all calves born during the specified time period that died on an agricultural holding within 365 days of birth.

In the logistic component of the models, the odds of a beef or dairy herd being closed decreased significantly as the calf mortality rate increased. Beef herds with average ages at first calving in the second and third quintiles (29.6 to 31.8 months and 31.9 to 33.8 months, respectively) were significantly less likely to be closed than herds in the top quintile (<29.6 months), while herds in the bottom quintile (>35.9 months) were significantly more likely to be closed. Similar trends with the average age at first calving were observed for dairy herds. Herds of both production types with culling rates above or below the industry target range were also significantly more likely to be closed. The average calving interval was not significantly associated with being a closed dairy herd. However, beef herds with calving intervals above the first quintile (>378 days) were generally less likely to be closed, although there was no clear trend as the calving interval increased. Herd size had no significant effect on the odds of either a beef or dairy herd being closed.

In the negative binomial component of the models, the total number of replacement breeding cattle purchased by beef and dairy herds generally increased with herd size, culling rate, and calf mortality rate. For dairy herds, there was also an increase in the number of replacement breeding cattle purchased as the average age at first calving increased. This trend was not observed for beef herds. For herds of both production types, having a calving interval in the bottom quintile (>412 days for beef and >444 days for dairy) was significantly associated with purchasing greater numbers of replacement breeding cattle.

**Figure 2:**
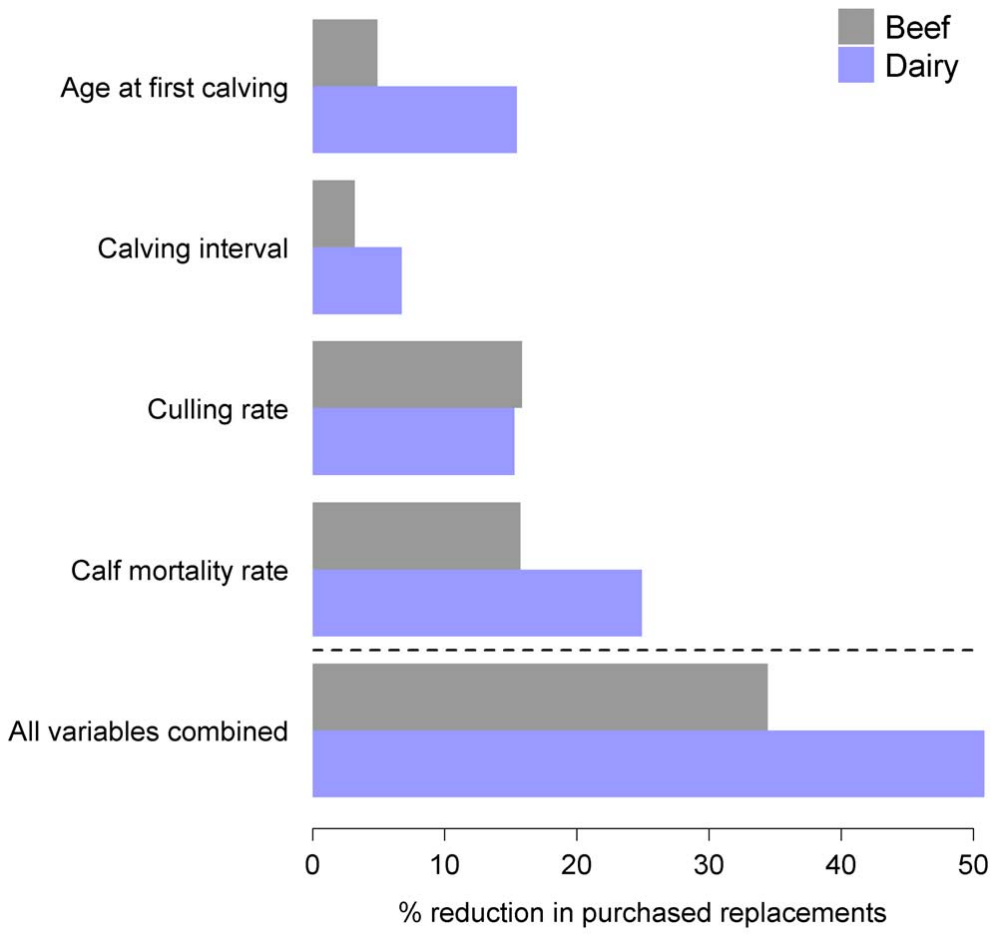
Estimated reduction in the number of purchased replacement breeding cattle with improved herd management. The horizontal bars show the percentage reduction in the total number of replacement breeding cattle purchased by the study herds when the values for age at first calving, calving interval, and calf mortality variables, the target value were set as the top quintiles and the values for culling rates were set at the middle quintile in the ZINB models. Each variable was tested alone and in combination.

The ZINB models were then used to predict the effects of altering herd performance on the total number of replacement breeding cattle purchased by the study herds (Figure 2). Setting all the reproductive performance variables for each herds to the top quintile reduced the number of replacement breeding cattle purchased by 34.4% for beef and 50.8% for dairy.

### Impact of replacement breeding cattle trade on disease transmission dynamics

**Figure 3:**
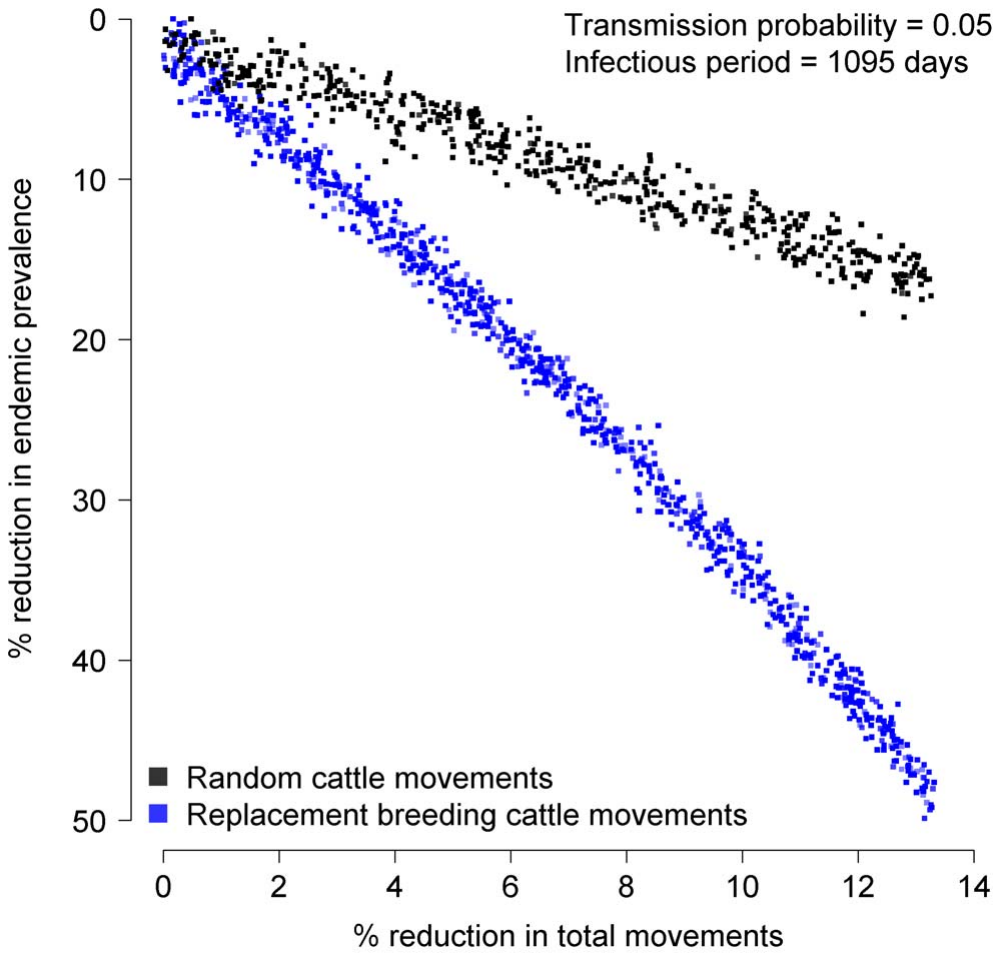
Estimated reduction in the endemic prevalence of BVDV following removal of replacement breeding cattle movements. The proportion of movements removed from the network was varied randomly between 0 and 13.3% at the beginning of each simulation. The black dots indicate the results from removing movements from the network at random. The blue dots indicate the results for the targeted removal of replacement breeding cattle movements. A total of 10,000 replicates were performed for each removal strategy. The transmission probability was set at 0.05 and the infectious period half-life was set at 1,095 days to simulate BVDV.

**Figure 4:**
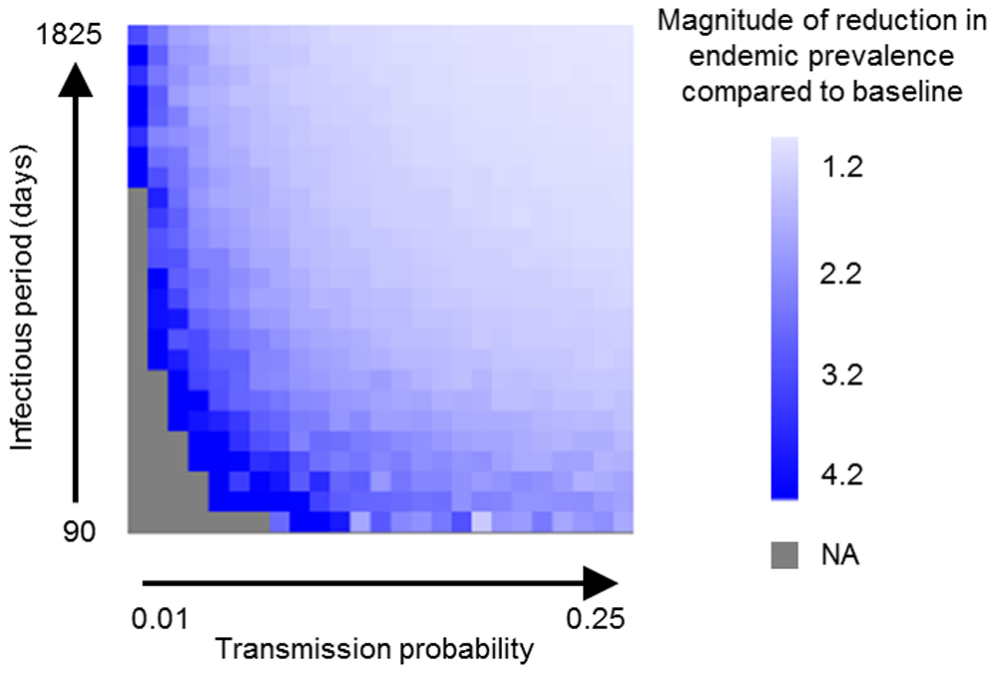
Effects of altering the transmission probability and infectious period half-life on simulation model results. The values shown are the predicted endemic prevalence when all replacement breeding cattle movements were removed from the network divided by the predicted endemic prevalence when an equivalent number of movements (including all movement types) were removed from the network at random. Grey squares indicate parameter combinations where disease was unable to persist on the network.

The simulation models revealed that replacement breeding cattle movements had a disproportionately strong influence on network transmission dynamics. At a transmission probability of 0.05 and infectious period half-life of 1,095 days, removal of all replacement breeding cattle movements (13.3% of all between-herd movements) from the network resulted in an approximately 45.8% reduction in endemic prevalence (Figure 3). Removal of the equivalent number of movements at random decreased endemic prevalence by only 19%. The effects of removing replacement breeding cattle movements compared to removing movements at random were more pronounced for diseases with low transmission probabilities and short infectious periods (Figure 4).

## Discussion

Although many studies have used records from the CTS database to investigate the spread of disease through cattle movement networks [20], [33], [39], [40], this is the first to our knowledge that establishes a direct relationship between the management practices of individual herds and the theoretical risk of infectious disease transmission. The most significant finding in the present study was that herds with poor performance were not only losing profitability, but also contributing to the persistence of endemic diseases at the industry level by purchasing excess numbers of replacement breeding cattle. The wide variation in performance between herds suggests that there is significant potential to reduce the number of replacement breeding cattle purchased and therefore the number of potentially infectious contacts by improving herd management. As a disease control strategy, this approach may be particularly effective because of the disproportionately strong influence that replacement trade has on the industry-level transmission dynamics of many important livestock pathogens

### Data limitations

There are several limitations in using the CTS database to calculate herd performance indicators that must be considered when interpreting the study findings. First, a breeding herd was defined as any location with a unique CPH number that had at least one recorded beef or dairy calving. Larger farm businesses may house cattle on several locations [41] and with the available data, it was not possible for us to determine which of these locations were linked. Therefore, some of the animals classified as replacement breeding cattle or culled cattle may have been transfers within the same farm business rather than transfers of ownership. We also assumed that dairy breeding cattle housed on the same location as beef breeding cattle were separate production units. However, these dairy cattle may have been strictly used to produce crossbreed calves for the beef production unit [42]. Second, farmers are not required to register the births of stillborn calves or calves that died within several hours of birth. The may lead to underestimation of calf mortality rates and breeding herd size as well as overestimation of the average age at first calving and calving intervals. Finally, records in the CTS database are not free from error and a small proportion of calving records were discarded due to missing or biologically implausible data.

### Impact of herd performance on replacement breeding cattle trade

Descriptive statistics revealed that the majority of beef and dairy herds in Great Britain were calving heifers at significantly older ages than the recommended 24 months [11]. However, the relationship between average age at first calving and the risk of purchasing replacement breeding cattle was complex. Compared to herds ranked in the top 20% for performance, those in second quintile were significantly more likely to be open, while those in the bottom quintile were significantly more likely to be closed. Part of this trend may related to the difficulty in ensuring that heifers have reached an appropriate physical maturity by the start of the breeding season or the target age at first calving for the herd. Heifers that are bred too young have a greater risk of calving complications [43], which can effect subsequent fertility and performance [44]. Consequently, farmers may choose to retain heifers for breeding in subsequent autumn or spring calving seasons [45], which would increase the average age at first calving, but reduce the need to purchase animals from outside sources. For dairy herds, the total number of replacement breeding cattle purchased increased with the average age at first calving. Based on unpublished data, this may be confounded by the fact that purchased replacement dairy heifers were also significantly older at the time of calving than home-raised heifers.

The average calving intervals observed in the study herds were also significantly greater than the recommended 365 days [42], [46], which suggests that many cattle breeding herds in Great Britain are experiencing problems with fertility. Delays between successive calvings should in theory limit the number of replacement heifers an animal produces over its lifespan leading to an increased risk of purchasing replacement cattle as well as an increased number of cattle purchased. However, contrary to expectations, the average calving interval had little appreciable effect on replacement breeding cattle trade. One possible explanation is that calving intervals may be artificially low in herds that are culling excessive animals for poor fertility [47], [48]. For example, beef herds that practice seasonal calving are under significant pressure to cull animals that fail to conceive within the narrow breeding window [32]. The results for dairy herds may also be confounded by the presence of high producing dairy herds that intentionally delay rebreeding in certain high yielding cows to increase farm profitability [49], [50]. Future studies should explore the interaction between the different performance variables in greater detail.

The risk of purchasing at least one replacement breeding animal was less in herds with culling rates that were above or below the industry target ranges. It is possible that some of the herds with low culling rates were compensating for an inadequate supply of replacement heifers by retaining a greater percentage of mature breeding cattle, while some of the herds with high culling rates were in the process of exiting the cattle industry. A small number of herds in England and Wales may have also been subject to movement restrictions and increased culling as part of bovine tuberculosis control efforts [51]. Even though the risk of disease introductions is theoretically lower, herds that cull too few animals are losing opportunities to improve herd genetics and performance, while herds that cull too many animals are losing profitability through the costs of raising extra replacement heifers to maintain herd size [9], [52]. The negative binomial portion of the ZINB models predicted that number of replacement breeding cattle increased with herd culling rates, which supports the hypothesis that herds with high culling rates have an increased demand for replacement cattle.

For herds of both production types, the total number of replacement breeding cattle purchased increased with the calf mortality rate. The magnitude of these results must be interpreted with some caution as the extent and effects of under-reporting the deaths of male calves are not well known [53]. Calf mortality has a direct impact on the supply of replacement heifers and it has been recommended that death losses should not exceed 5% [36]. The majority of beef herds were well below this threshold, which may explain why the risk of being a closed herd decreased only marginally as the calf mortality rate increased. In contrast, almost 60% of dairy herds had a mortality rate greater than 5%. This may be partly attributed to the fact that male dairy calves have a lower economic value and generally do not receive the same standard of care as replacement heifers [54]. Furthermore, dairy calves are separated from their dams shortly after birth and factors such as colostrum intake, housing conditions, nutritional management, and infectious disease control become even more critical in preventing calf deaths [55]–[57].

### Impact of replacement breeding cattle trade on disease transmission dynamics

In a recent review, Carslake and colleagues emphasized the importance of finding disease control interventions that are effective against a wide range of endemic diseases to reduce trade-off in resource allocation [58]. Our ZINB models predicted that if all herds were able to achieve the same level of performance as the top 20%, the number of replacement breeding cattle purchased by beef and dairy herds could be reduced by a third and a half, respectively. Given that even herds in the top 20% were still operating below industry targets for performance, these may be conservative estimates for the potential reduction in replacement breeding cattle movements and subsequent risk of introducing multiple directly transmissible diseases to the herd. The primary advantage of this approach is that improving herd performance has readily demonstrable effects on farm profitability without relying on disease specific interventions. There is still, however, the challenge of providing appropriate incentives and education to encourage farmers to change their management practices.

Poor performance has traditionally been considered a herd level problem and therefore free from national regulation. However, as the results from our simulation model show, the practice of purchasing replacement breeding cattle has a disproportionately strong influence on the risk of disease spreading to other farms in the network. This may be related to the market structure of the British livestock industry since herds that purchase replacement breeding cattle must often source animals from multiple herds, which increases the number of inward contacts. These farms may also be selling larger numbers of cattle for fattening, which increases the number of outward contacts. Both factors are important determinants of network centrality. Even if these movements cannot be prevented through improved herd management, it may possible to apply disease specific biosecurity measures such as quarantine, vaccination, or diagnostic testing to effectively remove them from the contact network [21], [59], [60]. However, these measures may be more effective against some pathogens than others. Our results also showed that the magnitude of the observed effect increased as both the farm infectious period and movement transmission probability were decreased. This is likely to be because diseases with short infectious periods and low transmission probabilities have difficulty persisting in cattle populations to begin with and therefore minor changes in the network structure are enough to push these diseases towards extinction. Other researchers have similarly shown that the structural and temporal features of cattle movement networks matter less for diseases that spread over long time periods [61] or have a higher probability of spreading through batch movements [33].

### Study limitations

Although we found many significant associations between herd performance and the purchase of replacement breeding cattle in the ZINB models, the interpretation of the study findings is complicated by the fact that poor performance can be both a cause and effect of purchasing replacement breeding cattle. For example, Thomsen and others [62] found that culling rates were significantly higher in Danish dairy herds with a large proportion of purchased cows. It was suggested that herds with excessively high culling rates may not have an adequate supply of heifers to meet replacement needs thereby necessitating the purchase of replacement breeding cattle from outside sources. However, herds that purchase replacement breeding cattle are at increased risk of introducing diseases like bovine viral diarrhoea virus (BVDV) and bovine herpesvirus type I (BHV I), which can in turn lead to increased culling through their effects on fertility and abortion [63]–[66]. Similarly, high calf mortality rates may limit the availability of replacement heifers, but may also be linked to the presence of infectious diseases introduced through animal movements [67], [68].

Another striking feature of our results was the number of herds operating below industry standards for animal performance. The potential causes of poor performance in British beef and dairy herds have been discussed at length in a previous publication [29] and are likely farm specific and multifactorial. This leads to the question of how much farmers can reasonably be expected to improve herd performance in the field. Our models assumed that all herds would be capable of achieving the same level of performance as the top 20% of herds in the field, which in many cases was still below the industry targets for performance. Further research is needed to determine whether this leads to an underestimation or overestimation of the effect size observed in the ZINB models.

The simulation study used a simplistic disease transmission model that considered all farms to be homogenous production units regardless of their size or demographic structure and all movements to carry the same risk of transmitting disease regardless of the number or production type of cattle moved. While these assumptions may be appropriate for highly infectious epidemic diseases that spread rapidly and indiscriminately between herds, endemic pathogens often have unique epidemiological features that can modify transmission risk [58]. For example, factors such as age, gender, and production type can influence the probability of purchased cattle being infected as well as their probability of being commingled directly with susceptible production groups in the receiving herd [69]. The rate of disease clearance from infected herds can also be influenced by size and other management practices [70], [71]. We also assumed that no disease transmission occurred between animals in close contact at livestock markets, which again may change the industry level transmission dynamics. Therefore, the absolute values predicted by the model should be interpreted with caution, but the general trend that replacement breeding cattle movements have a greater importance to disease transmission should still be robust.
